# Supplementary material for: Indisulam synergizes with palbociclib to induce senescence through inhibition of CDK2 kinase activity
Source: PLoS One. 2022 Sep 6;17(9):e0273182. doi: 10.1371/journal.pone.0273182 (PMC9447877; doi:10.1371/journal.pone.0273182)

Supplemental Figure 1

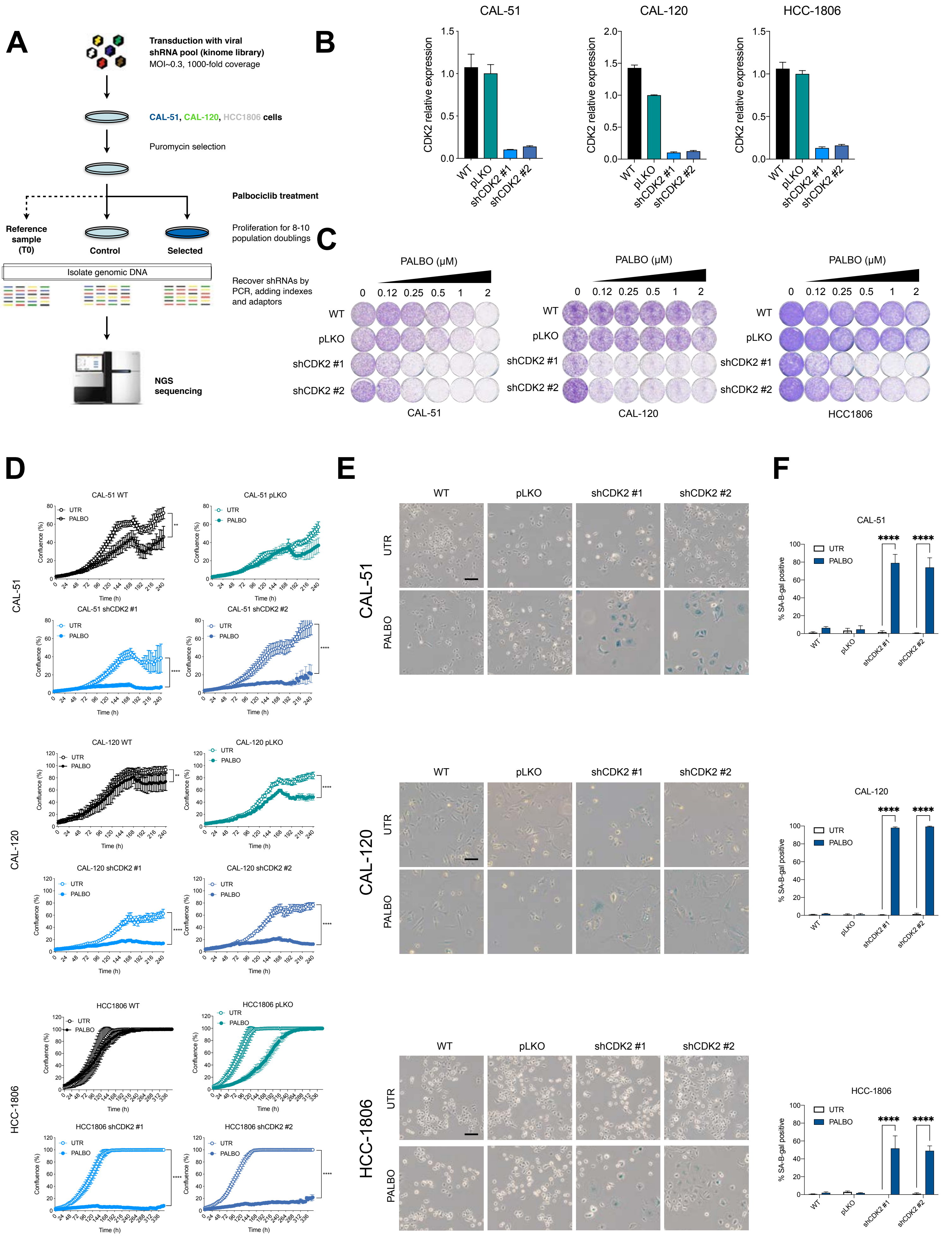

Supplemental Figure2

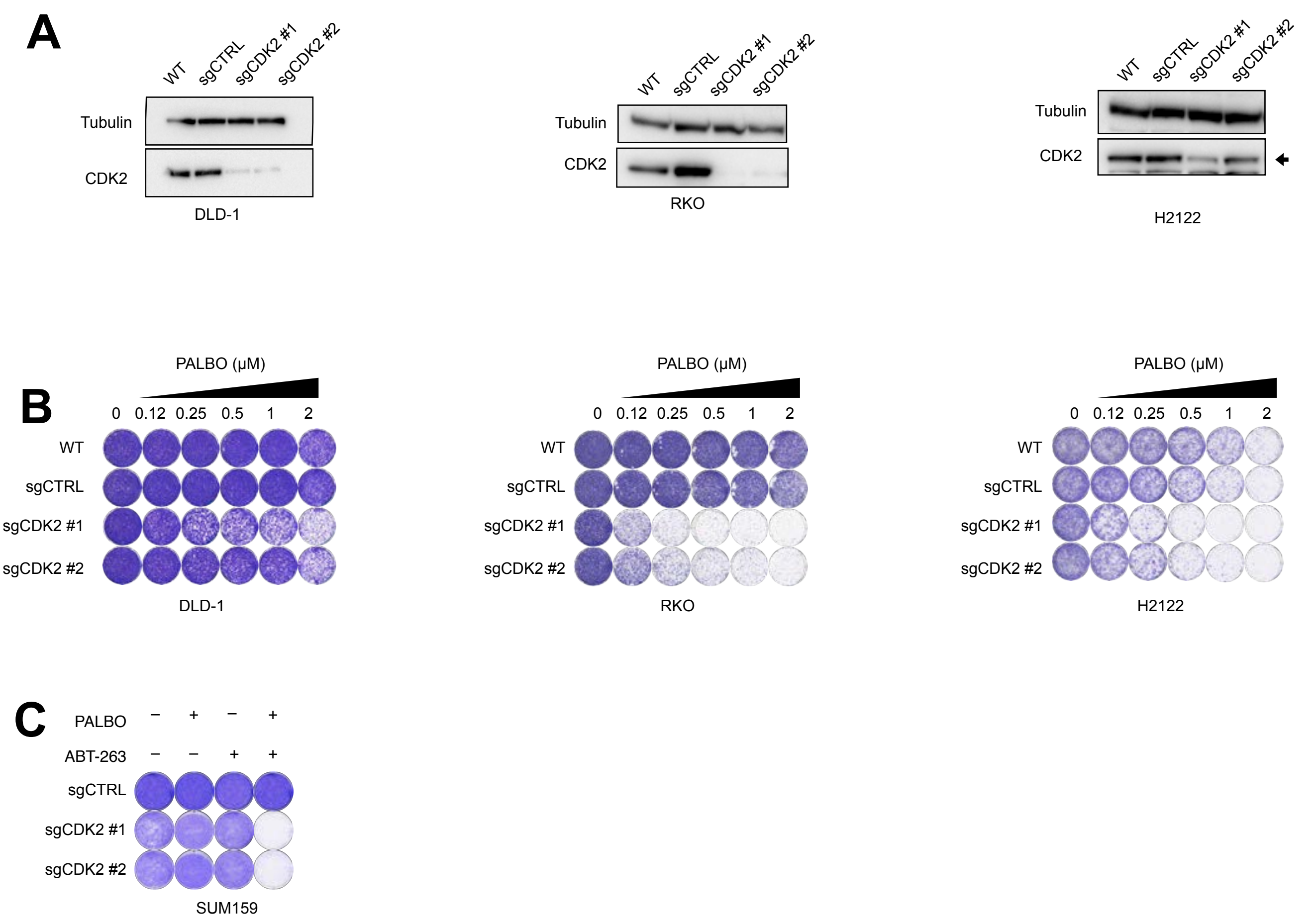

Supplemental Figure 3

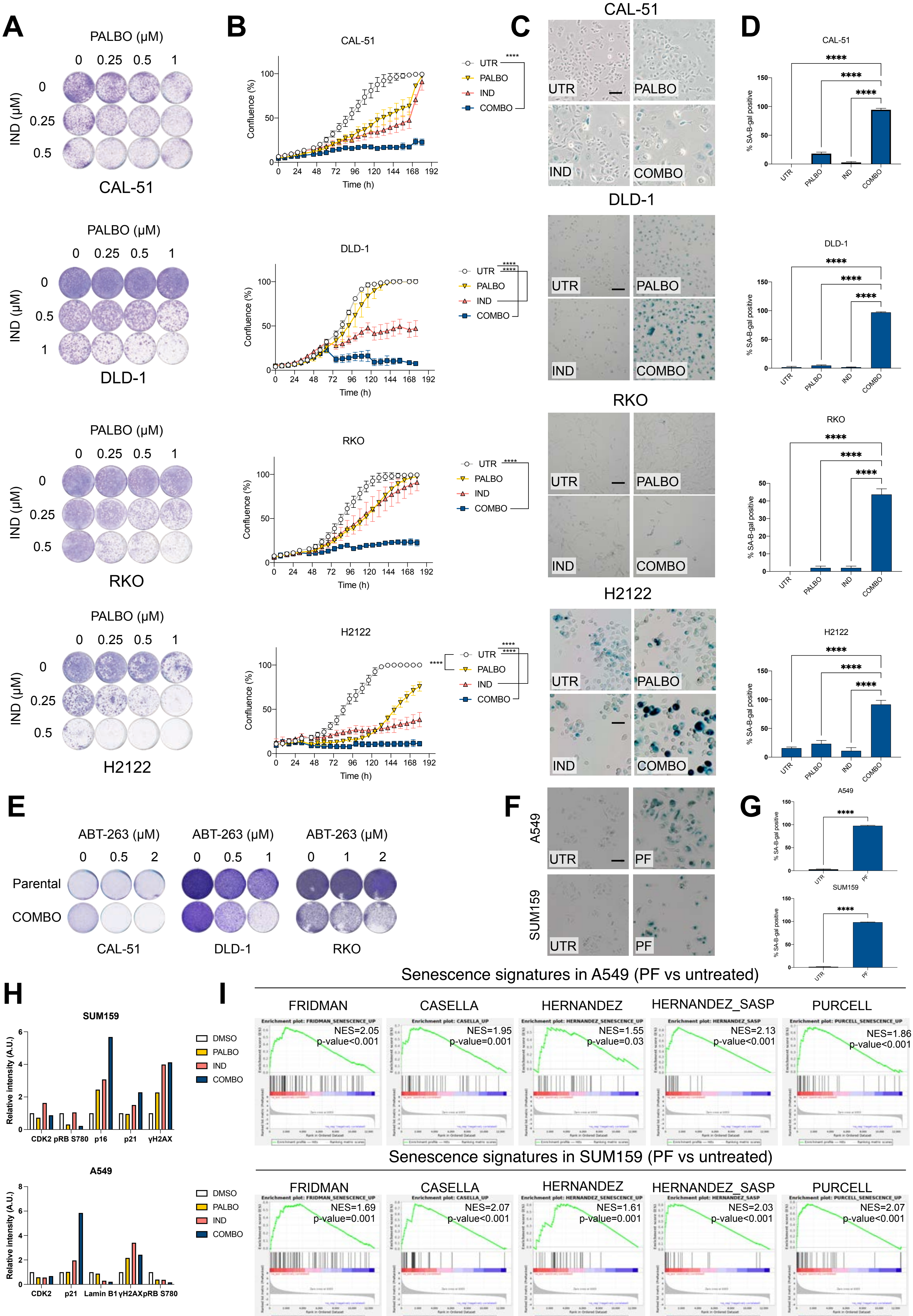

Supplemental Figure 4

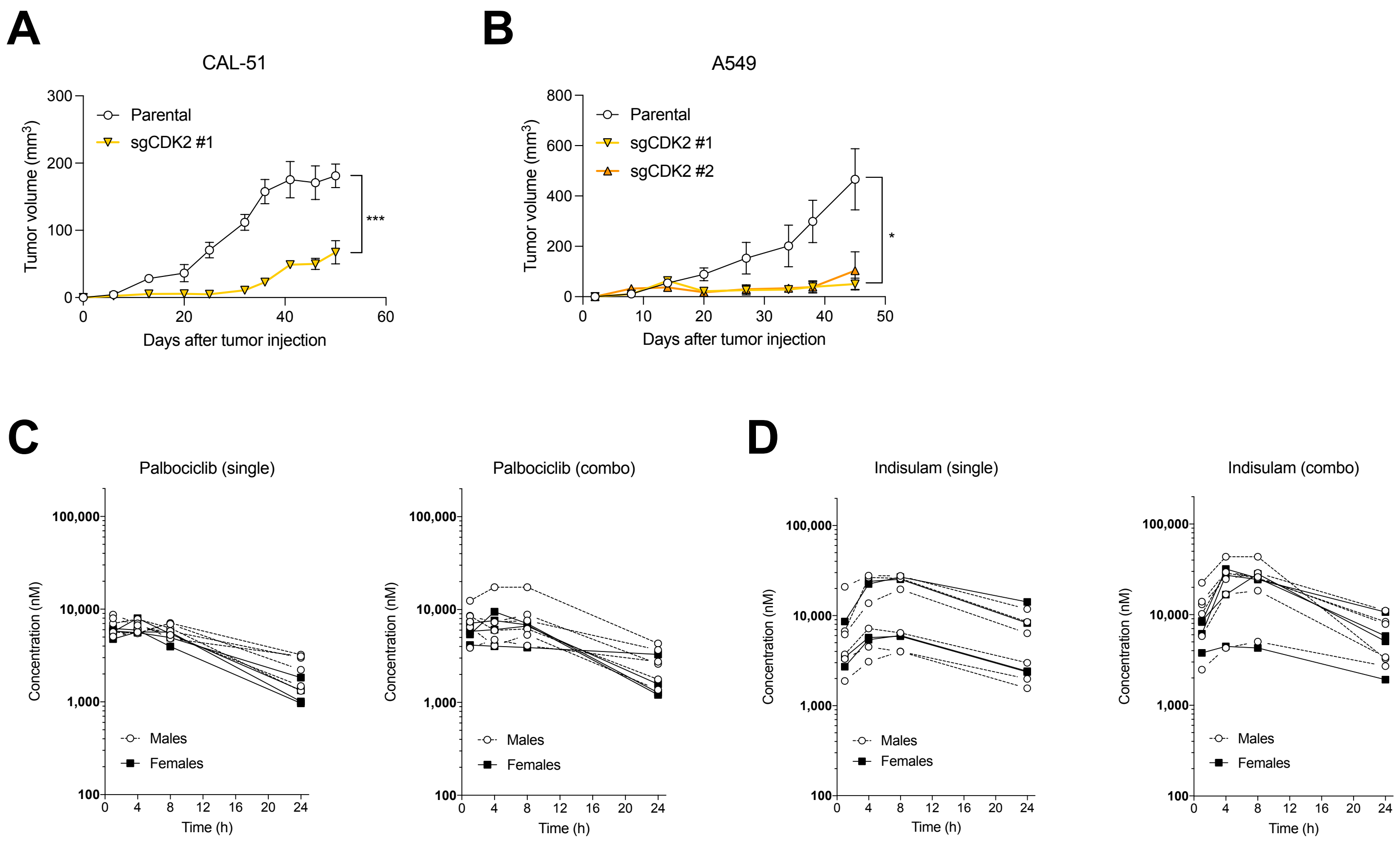

Supplement Figure 5

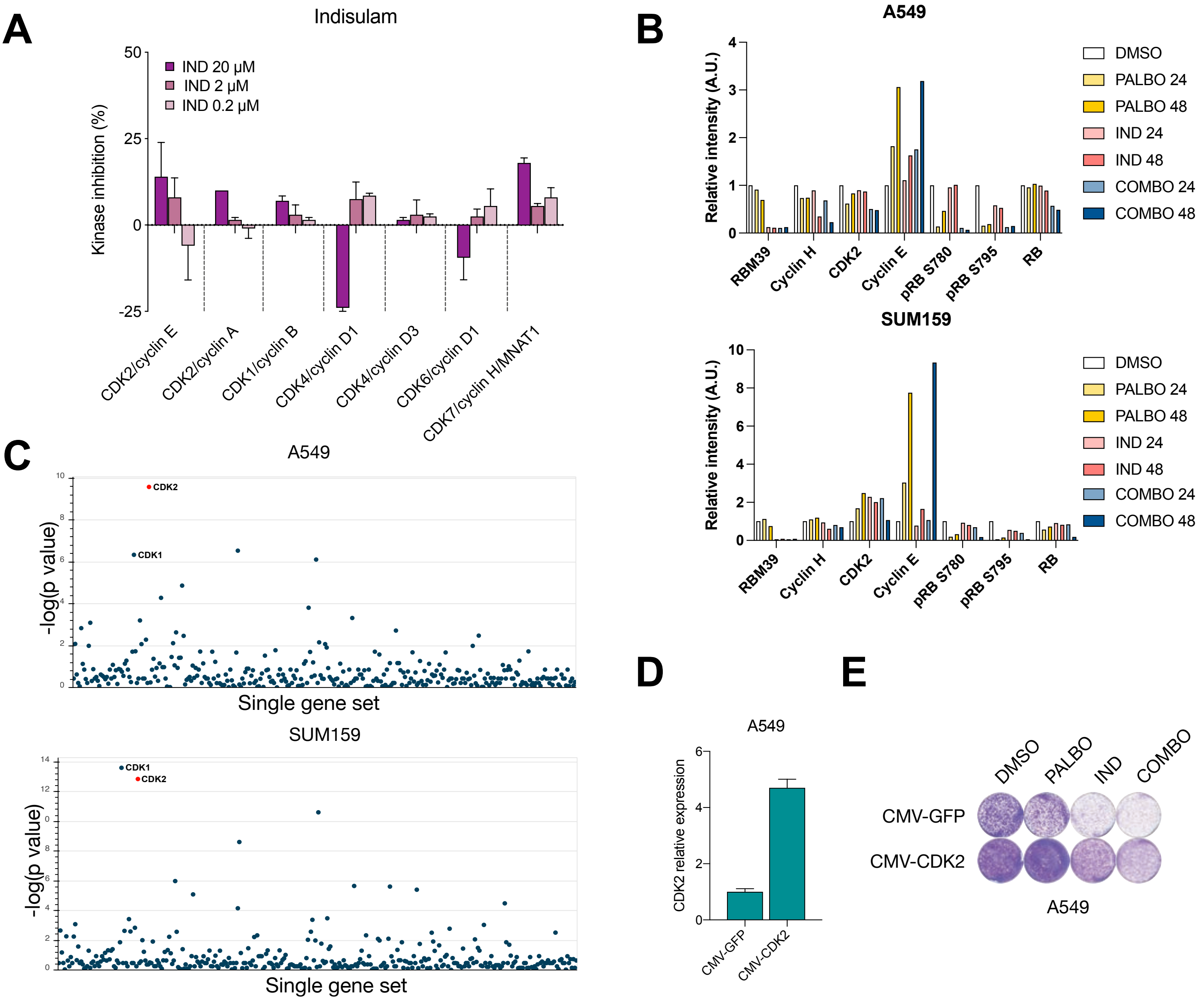

Supplement: S1 File — (PDF) [file pone.0273182.s001.pdf]
