# Supplementary material for: Indisulam synergizes with palbociclib to induce senescence through inhibition of CDK2 kinase activity
Source: PLoS One. 2022 Sep 6;17(9):e0273182. doi: 10.1371/journal.pone.0273182 (PMC9447877; doi:10.1371/journal.pone.0273182)
Supplement: S2 File — (PDF) [file pone.0273182.s002.pdf]

Supplement Figure 6

A

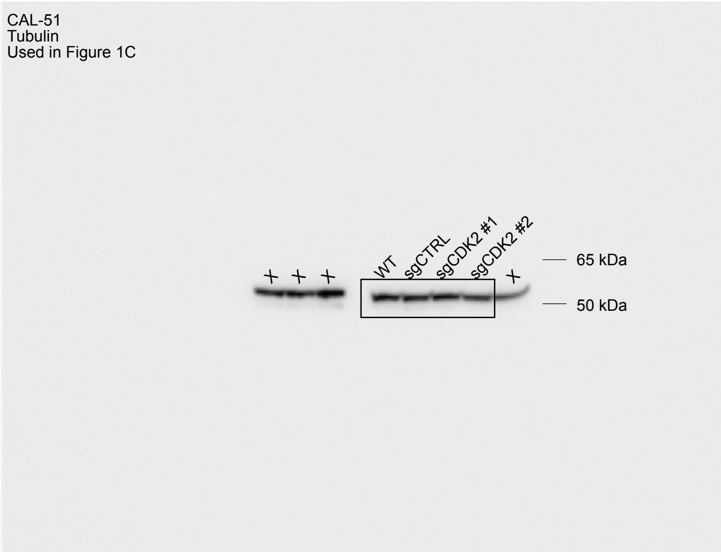

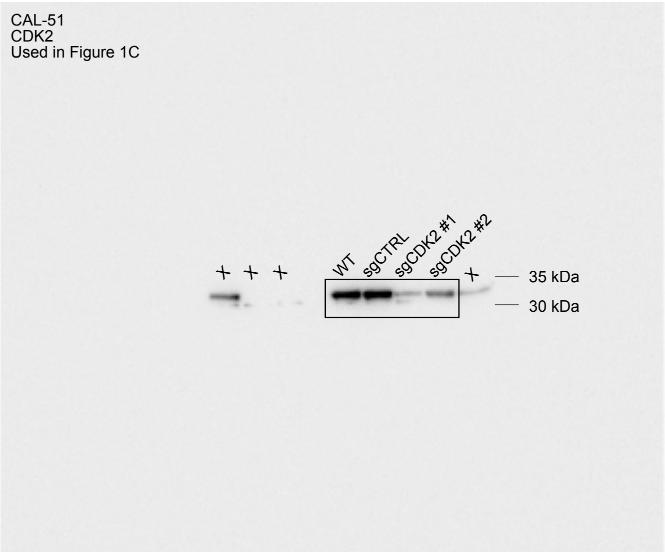

B

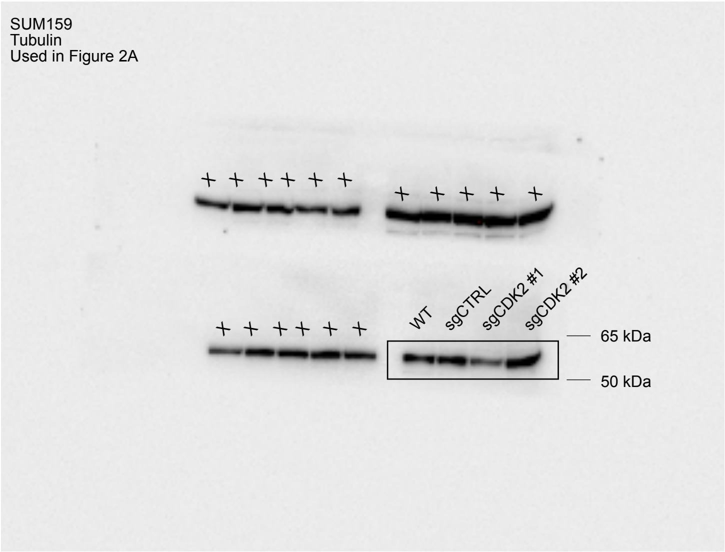

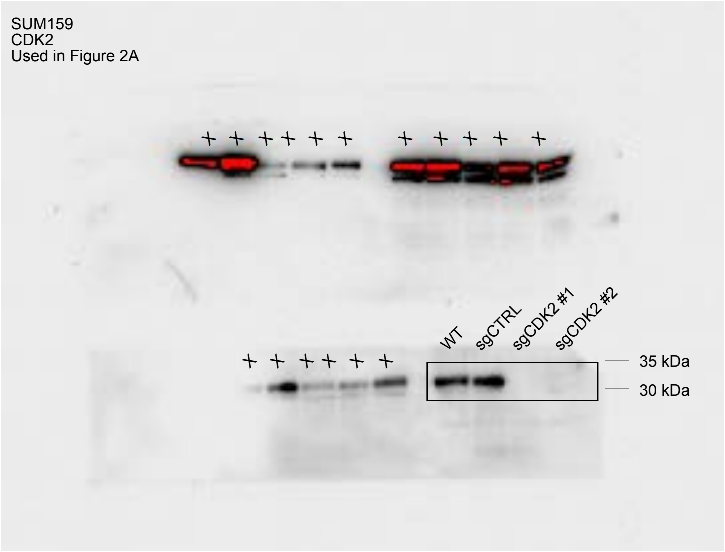

C

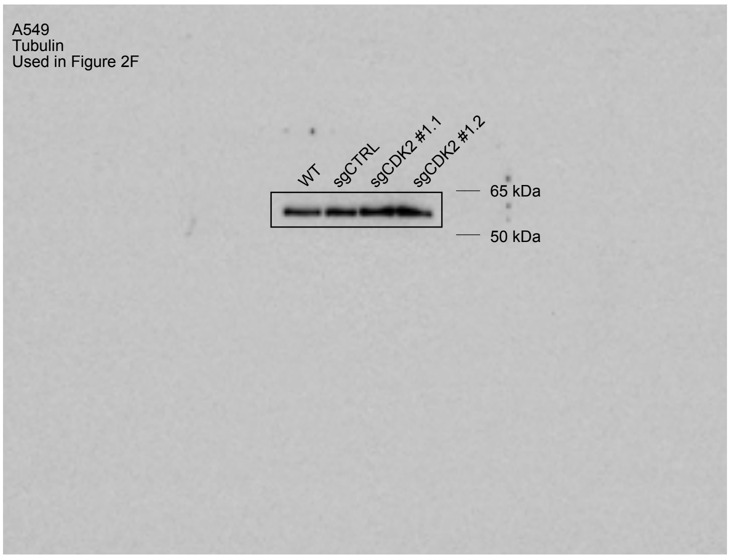

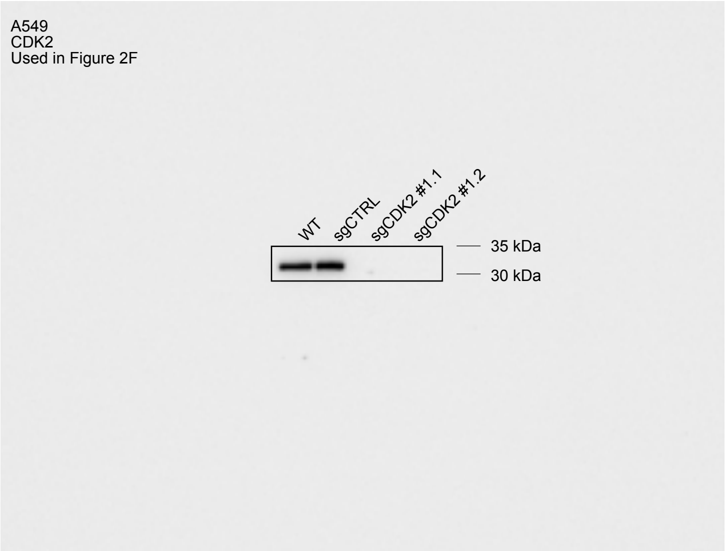

D

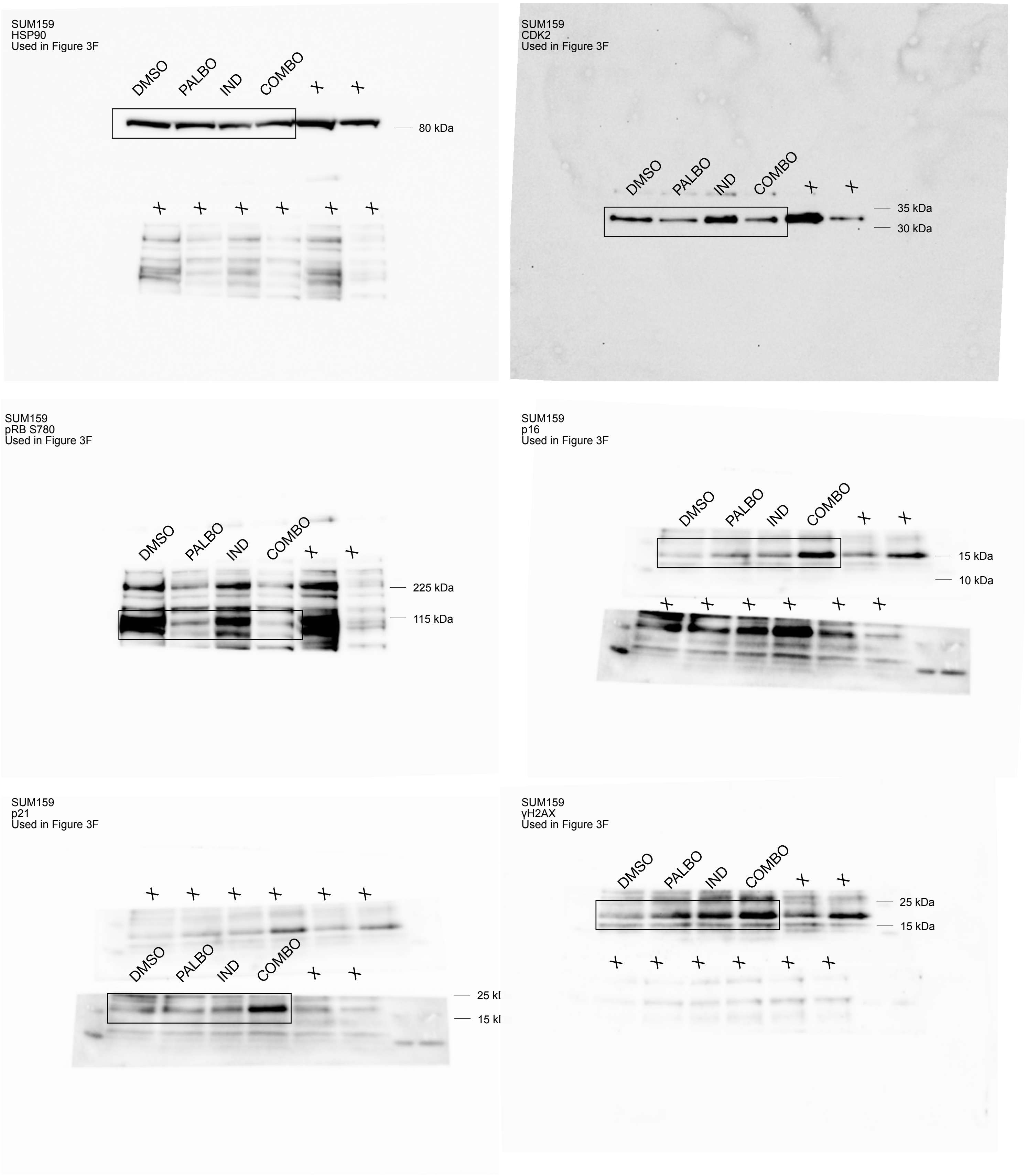
SUM159
HSP90
Used in Figure 3F
DMSO
PALBO
IND
COMBO
80 kDa
SUM159
CDK2
Used in Figure 3F
DMSO
PALBO
IND
COMBO
35 kDa
30 kDa
SUM159
pRB S780
Used in Figure 3F
DMSO
PALBO
IND
COMBO
225 kDa
115 kDa
SUM159
p16
Used in Figure 3F
DMSO
PALBO
IND
COMBO
15 kDa
10 kDa
SUM159
p21
Used in Figure 3F
DMSO
PALBO
IND
COMBO
25 kI
15 kI
SUM159
γH2AX
Used in Figure 3F
DMSO
PALBO
IND
COMBO
25 kDa
15 kDa

A549
Vinculin
Used in Figure 3F

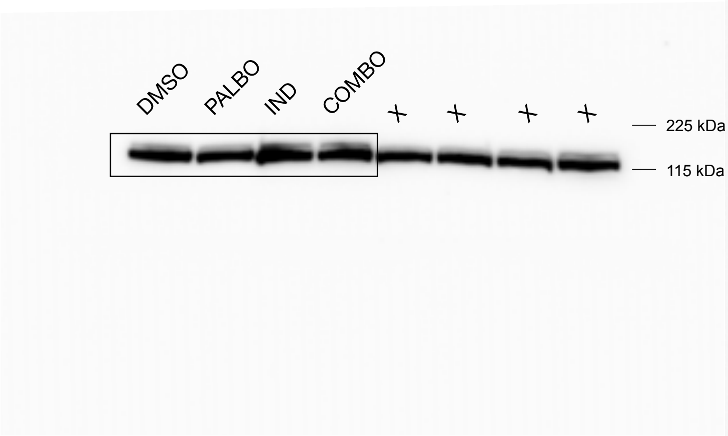

A549
CDK2
Used in Figure 3F

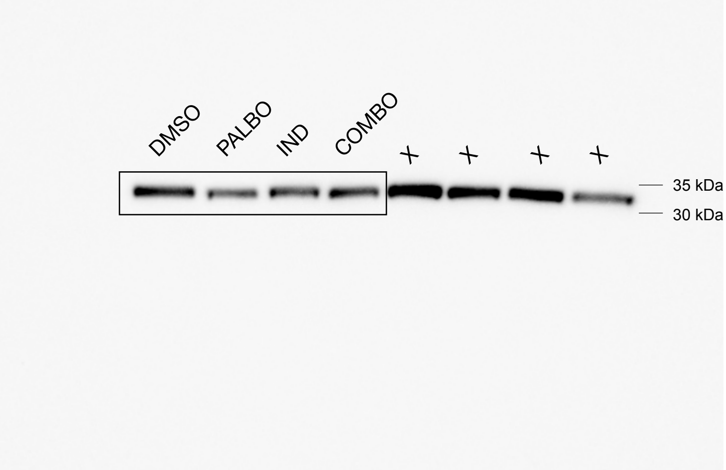

A549
P21
Used in Figure 3F

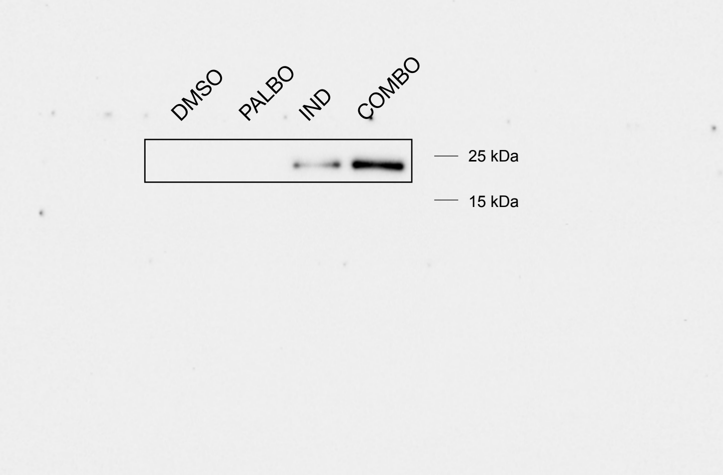

A549
Lamin B1
Used in Figure 3F

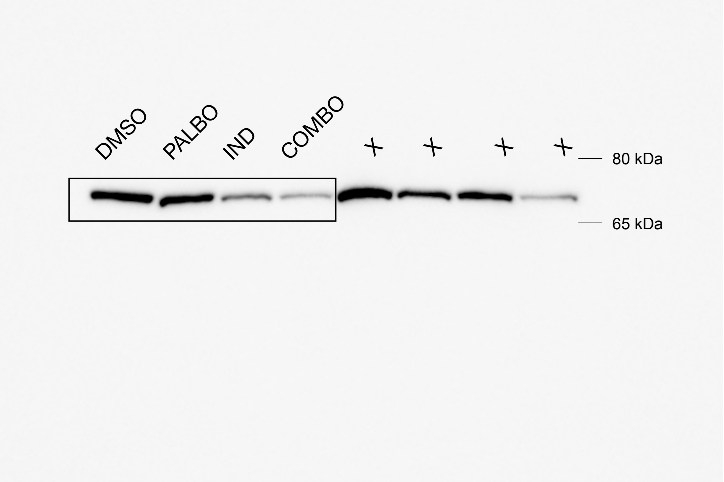

A549
γH2AX
Used in Figure 3F

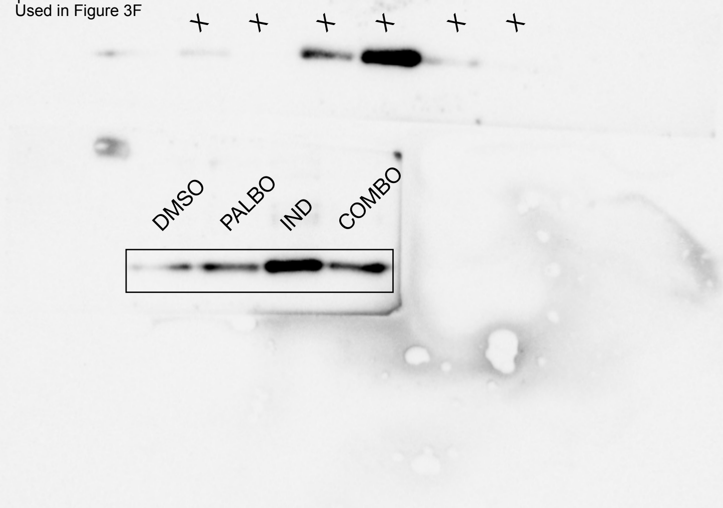

A549
Tubulin
Used in Figure 3F

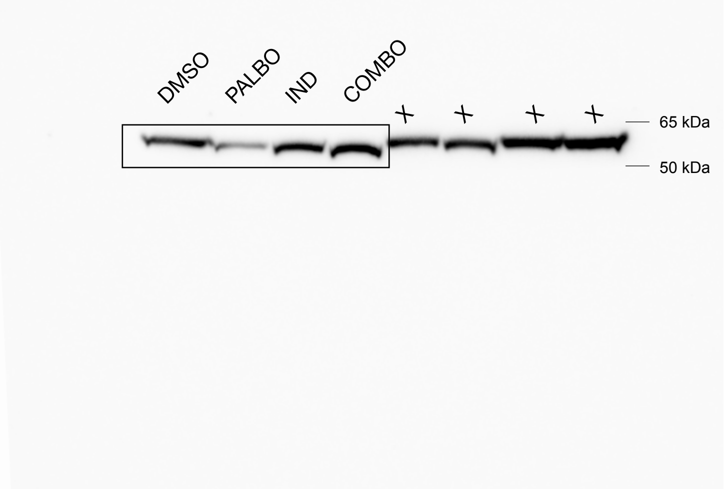

A549
pRB S780
Used in Figure 3F

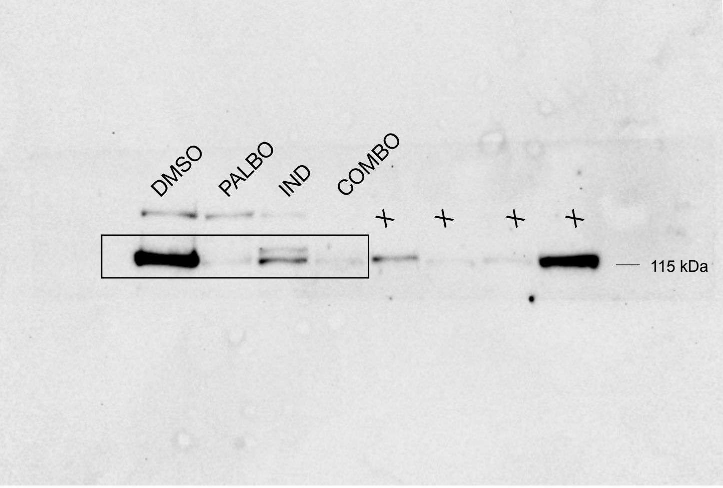

E

A549 and SUM159
Vinculin
Used in Figure 5A

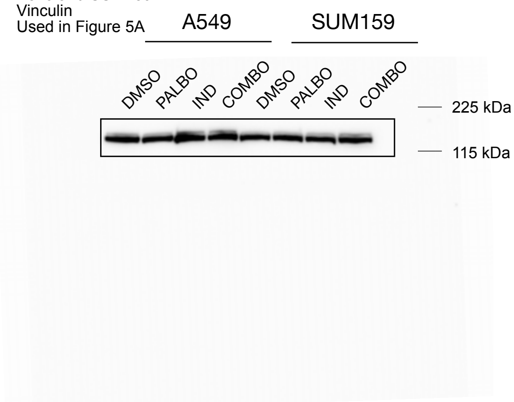

A549 and SUM159
RBM39
Used in Figure 5A

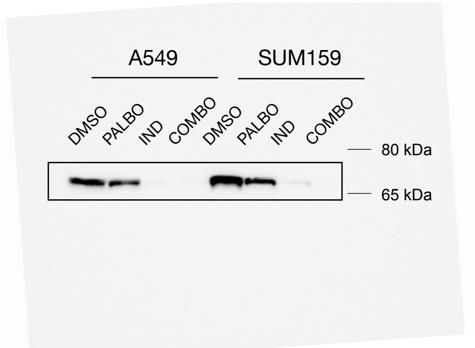

F

A549
HSP90
Used in Figure 5J

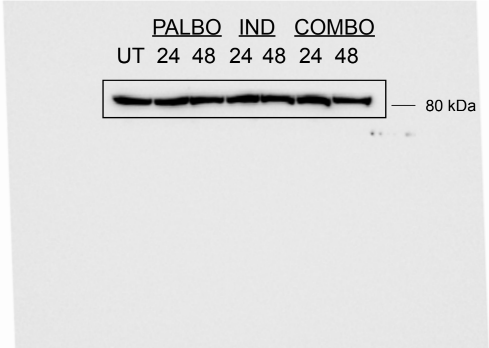

A549
RBM39
Used in Figure 5J

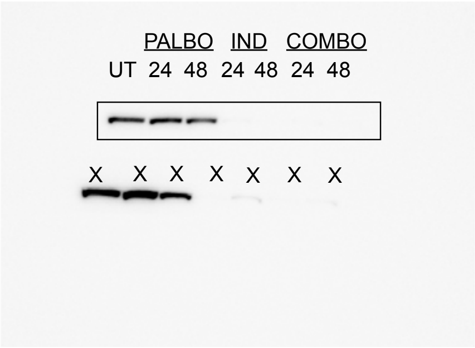

A549
Cyclin H
Used in Figure 5J

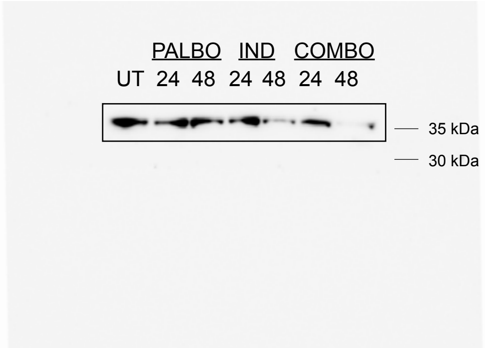

A549
CDK2
Used in Figure 5J

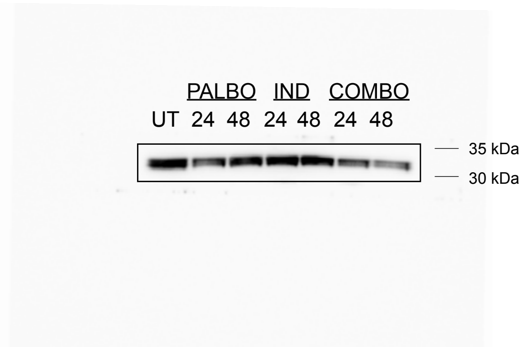

A549
Cyclin E
Used in Figure 5J

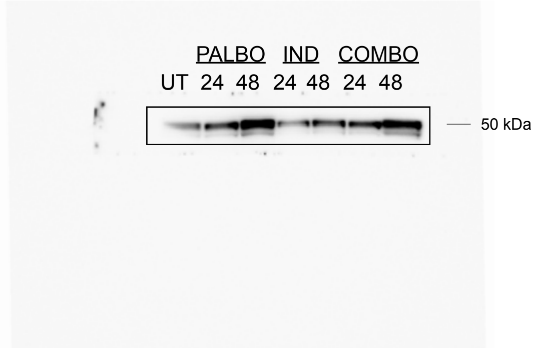

A549
pRB S780
Used in Figure 5J

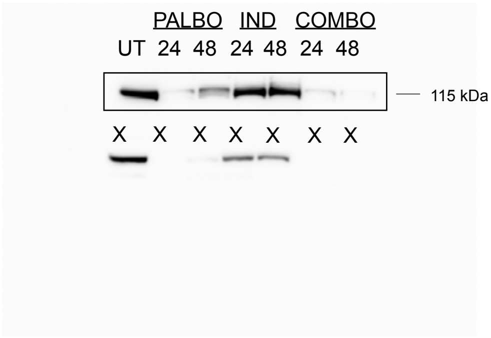

A549
pRB S795
Used in Figure 5J

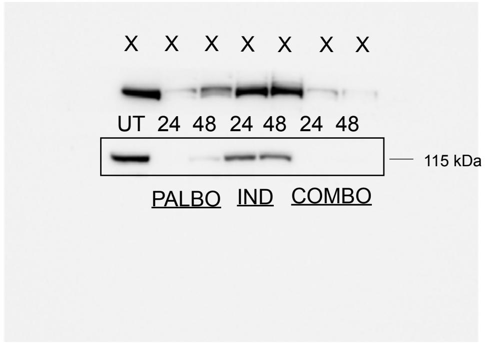

A549
RB
Used in Figure 5J

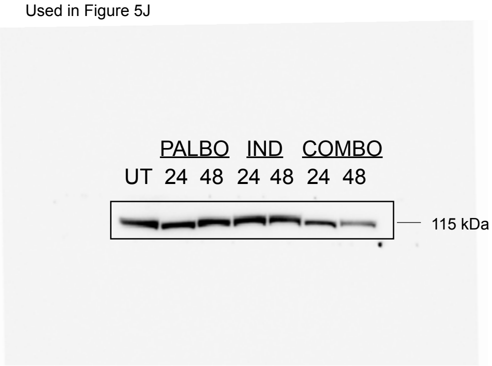

F

SUM159
HSP90
Used in Figure 5J

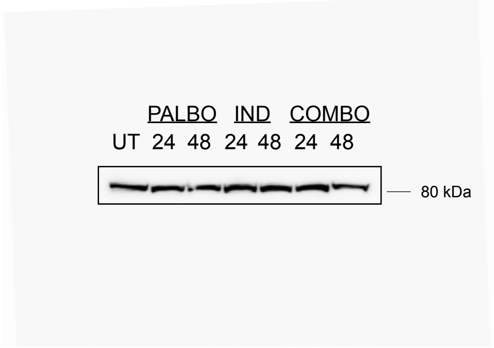

SUM159
RBM39
Used in Figure 5J

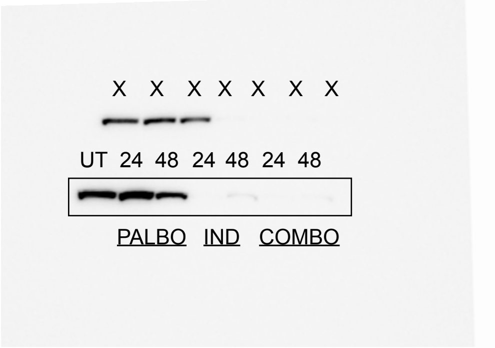

SUM159
Cyclin H
Used in Figure 5J

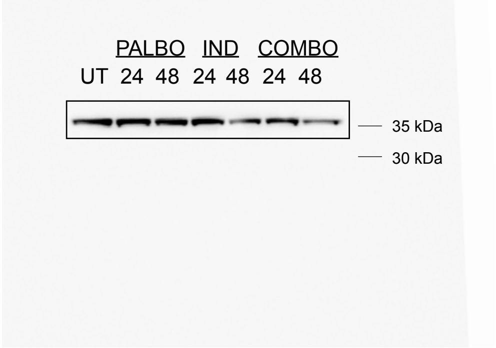

SUM159
CDK2
Used in Figure 5J

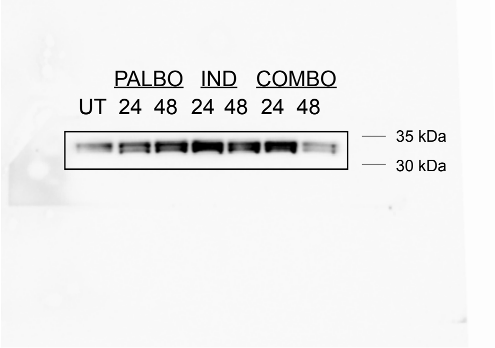

SUM159
Cyclin E
Used in Figure 5J

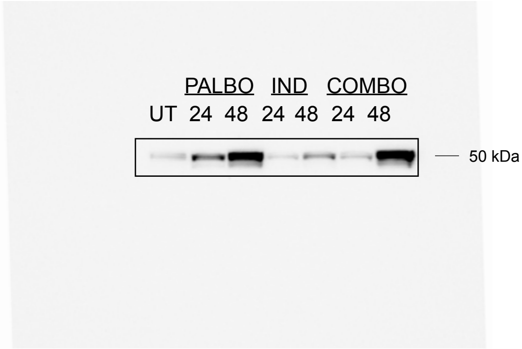

SUM159
pRB S780
Used in Figure 5J

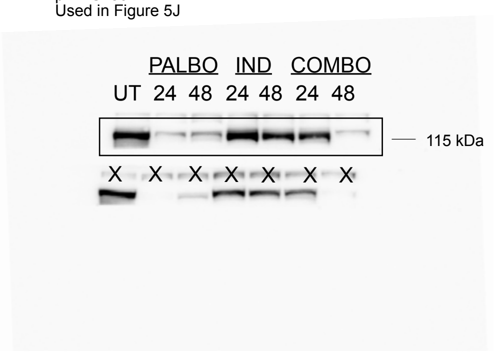

SUM159
pRB S795
Used in Figure 5J

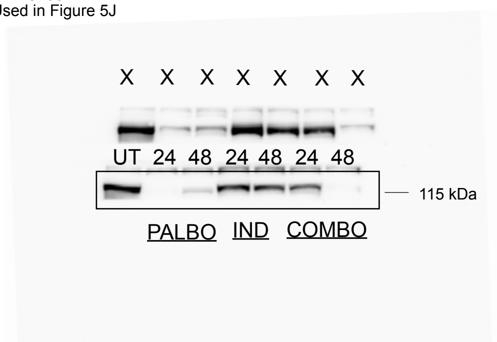

SUM159
RB
Used in Figure 5J

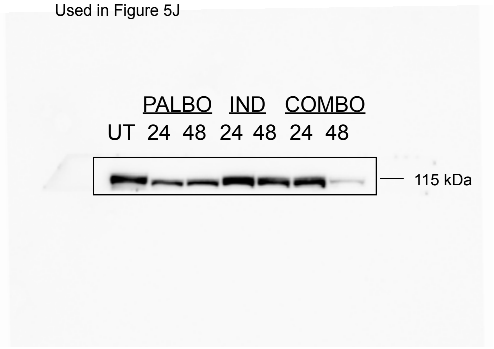

G

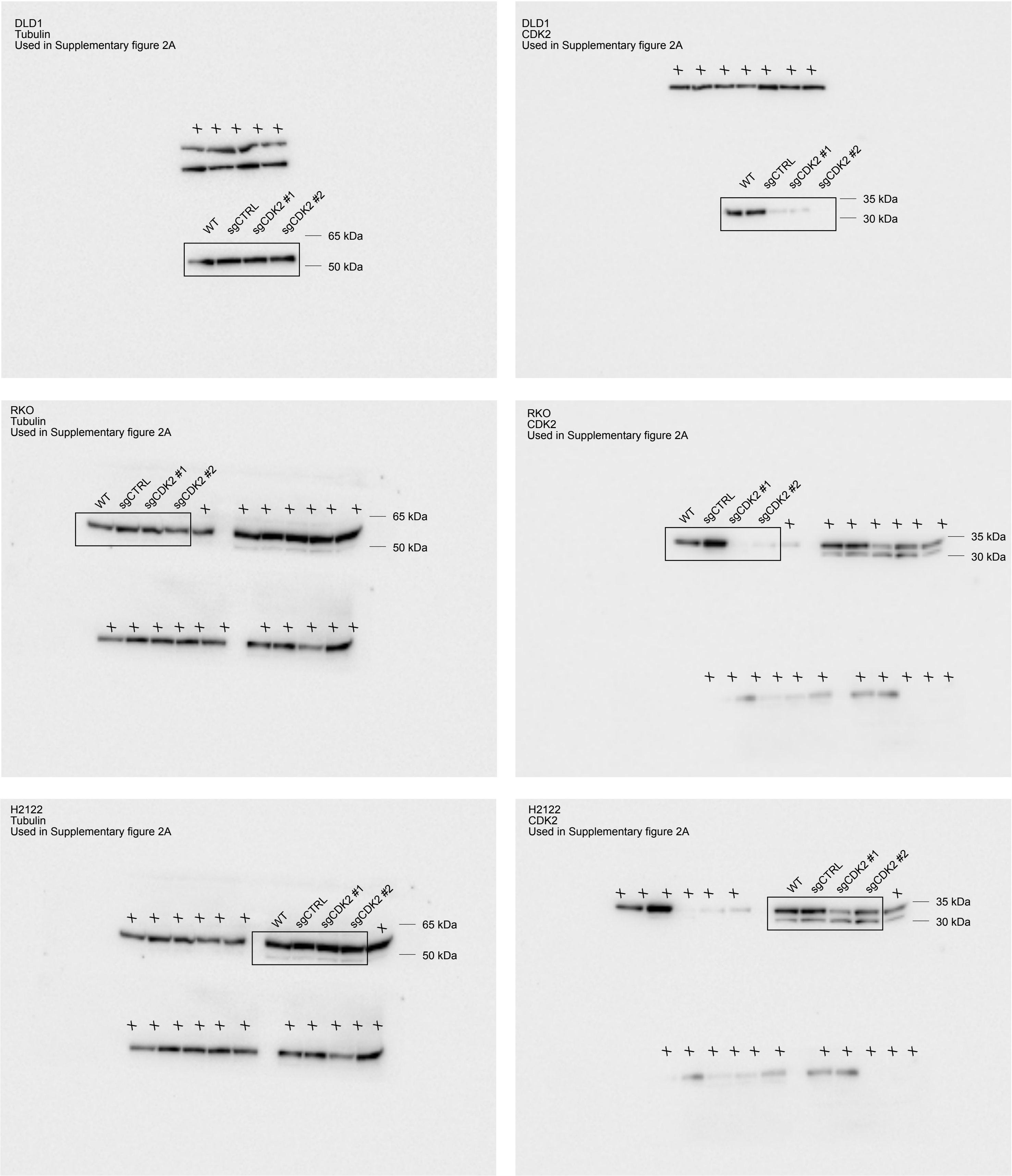
DLD1
Tubulin
Used in Supplementary figure 2A
WT
sgCTRL
sgCDK2 #1
sgCDK2 #2
65 kDa
50 kDa
DLD1
CDK2
Used in Supplementary figure 2A
WT
sgCTRL
sgCDK2 #1
sgCDK2 #2
35 kDa
30 kDa
RKO
Tubulin
Used in Supplementary figure 2A
WT
sgCTRL
sgCDK2 #1
sgCDK2 #2
65 kDa
50 kDa
RKO
CDK2
Used in Supplementary figure 2A
WT
sgCTRL
sgCDK2 #1
sgCDK2 #2
35 kDa
30 kDa
H2122
Tubulin
Used in Supplementary figure 2A
WT
sgCTRL
sgCDK2 #1
sgCDK2 #2
65 kDa
50 kDa
H2122
CDK2
Used in Supplementary figure 2A
WT
sgCTRL
sgCDK2 #1
sgCDK2 #2
35 kDa
30 kDa
