# Supplementary material for: Indisulam synergizes with palbociclib to induce senescence through inhibition of CDK2 kinase activity
Source: PLoS One. 2022 Sep 6;17(9):e0273182. doi: 10.1371/journal.pone.0273182 (PMC9447877; doi:10.1371/journal.pone.0273182)
Supplement: Attachment — Submitted filename: 120324_1_rebuttal_2203608_r168zd.docx [file pone.0273182.s004.docx]

**Manuscript number:** RC-2021-01047

**Corresponding author(s):** Rodrigo Leite de Oliveira, Rene Bernards

## 1. General Statements

We would like to thank all three reviewers for their feedback and constructive comments. We were pleased to read that the reviewers feel that our findings are relevant to the field and we hope you will find that the plan for revision adequately addresses the reviewers’ comments. All changes in the manuscript are marked by blue color.

## 2. Description of the planned revisions

**Reviewer 1, major comment 1:**

The concentrations of palbociclib and indisulam treatments in this whole study are obviously inconsistent even on the same cell line. For the palbociclib treatment on CAL-51, the author used 0.4 uM for shRNA screening, 0.5 uM for in vitro cell growth and SA-b-Gal, 2 uM for ABT-263 assay, and 2.5 uM for RNA-seq. It's required to unify them or provide a reasonable explanation of how the author decided treatment conditions.

We agree with the reviewer that concentrations differ between the experiments. We decided to remove the RNAseq data for CAL-51 as it is indeed less informative due to the concentration used. Furthermore, we will repeat the ABT-263 experiment using a lower dose of palbociclib to make it more comparable to the other experiments.

**Reviewer 1, major comment 3:**

In Fig. 1D, WT and sgCTRL CAL-51 cells showed the reduced colony amount under 2 uM palbociclib treatment, which implied cell death or cell cycle arrest. Although the author mentioned that CDK2 lacking cells showed senescence phenotypes (the increased SA-b-Gal positive ratio) under 0.5 uM palbociclib treatment compared with sgCTRL cells, there is no evidence to support the different senescence induction rate under 2 uM of treatment. It's quite difficult to understand why ABT-263 cannot kill the 2 uM palbociclib pre-treated sgCTRL cells in Fig. 1H. Please provide the SA-b-Gal staining results of 2 uM palbociclib treated sgCTRL and sgCDK2 CAL-51 cells.

This comment is similar to comment 1. We agree that the RNAseq experiment is not informative due to the high concentration of palbociclib used and we decided to remove it. We will repeat the ABT-263 experiment using lower concentration of palbociclib for senescence induction so that it is comparable to other experiments.

**Reviewer 1, minor comment 6:**

The representative images of Fig. 5D were unclear. Please provide quantitative data and statistical inferences for all SA-b-Gal staining results.

We agree that it would be beneficial to include the quantification of the SA-B-gal stainings. We will include this analysis in the revised manuscript.

**Reviewer 2, major comment 4:**

Fig S3C: Palbociclib treatment shows a very pronounced SA ß gal production -which is in contrast to Fog S1E. Why is that? Please discuss this.

This is a very interesting observation. The experiments were done in a slightly different way, cells in S1E were treated with 0.5µM palbociclib and cells in S3C with 1µM. Furthermore, cells were re-seeded before staining in S1E and not in S3C. We will repeat the S3C experiment in a way that matches the conditions of S3C and include it in the revised manuscript.

**Reviewer 2, major comment 7:**

Fig 5D: The ßgal staining does not look very convincing. How are the p16 and p21 protein levels?

We agree with the reviewer. These experiments were technically challenging, because RBM39 is an essential gene. Partial knockdown of RBM39 induced senescence in cells treated with palbociclib, but we would not expect this to happen in all cells due to heterogeneity of the knockdown. We will quantify the results of the SA-B-gal staining and include that in the revised manuscript.

**Reviewer 3, minor comment 3:**

The authors claim that navitoclax/ABT-263 induces cell death in palbociclib+indisulam treated cells however there is not actual proof of cell lethality. The experiments shown: colony growth and cell proliferation do not evaluate cell viability and thus the results are compatible with both cell proliferation and cell death.

We agree with the reviewer that it would be insightful to evaluate apoptosis after ABT-263 treatment. We will perform this experiment and add it to the revised manuscript.

**Reviewer 3, minor comment 4:**

In experiment 1H CAL-51 "cells were treated with 2 mM palbociclib for 10 days to induce senescence". According to figure 1D this treatment inhibits colony formation which is compatible with senescence induction. However, these senescent cells are insensitive to navitoclax (!). A SA-bgal assay showing the effect of palbociclib at a 2 mM concentration in control and sgCDK2 cells before ABT treatment ("palbo pre-treated") will help to understand these experiments.

This comment is similar to the major comment 1 and 3 from Reviewer 1. We agree with the reviewer that this result is surprising. We will repeat this experiment with a lower dose of palbociclib, to make it comparable with other experiments.

## 3. Description of the revisions that have already been incorporated in the transferred manuscript

**Reviewer 1, major comment 2:**

On page 6 lines 111-114, the author mentioned that CDK2 knockdown did not affect proliferation but only resensitized cells to palbociclib treatment. Please make statistical inferences to support this claim and all other cell growth results.

Comparing the untreated conditions of WT and CDK2 KO or KD cells in Figures 1D, 1E, S1C and S1D show no effect of CDK2 loss on viability. We performed statistical tests on the end point of all the growth curve experiments and added it to the figures and figure legends in the revised manuscript. Additionally, we compared the growth curves of the untreated conditions of WT and CDK2 KO CAL-51 below and did not observe significant differences in proliferation.

**Reviewer 1, major comment 4:**

In the figure legend of Fig. 1H, the author mentioned "... induce senescence, then seeded in high density and treated with ...". Please scientifically describe what the "high density" means.

We agree with the reviewer that the term ‘high density’ could be vague. We adjusted the text to say 100% confluence instead.

**Reviewer 1, major comment 5:**

In Fig. 1G, the author used gene sets from other previous studies, such as ref. 38-40, which were not recorded by Msigdb. Both Casella and Hernandez identified multiple senescence-related gene sets in different approaches. The author should describe the contents of gene sets in more detail.

The reviewer is correct that there are multiple gene sets present in Casella and Hernandez papers. We added a supplementary table 1, which shows exactly which gene sets were used for the analysis and mentioned the table in the methods section.

**Reviewer 1, major comment 6:**

According to Fig. 1H, because ABT-263 targets to Bcl-2 family, the phenotype of anti-apoptosis is supposed to be observed in 2 uM palbociclib treated sgCDK2 CAL-51 cells. Please provide the apoptosis-related and senescence_down GSEA results in Fig. 1G.

We removed this RNAseq data from the manuscript since it is less informative due to concentration of palbociclib used (see reply to major Reviewer 1’s major comment 1 and 3).

**Reviewer 1, major comment 8:**

In Fig. S3B, the cell numbers of DLD-1 cells suddenly decreased at 72 hr under double treatments of palbociclib and indisulam. If there was no technical problem, the author must further explain this phenomenon.

This is an interesting observation. The culture medium was refreshed at 72h which corresponds with decrease of confluence in the combination and indisulam treatment. Furthermore, this is a time point where we would expect senescence induction and the observed decrease in confluency can be due to changes in cell morphology when the cells enter senescence thus slightly impacting the technical detection of cell density.

**Reviewer 1, major comment 9:**

According to Fig. 2H and 3C, the higher concentration of palbociclib (2 uM) was enough to induce senescence in A549 cells. The synergic effect of combined treatment was unclear here.

We are not entirely sure what reviewer means, as figure 2H was performed with 0.5µM of palbociclib. We agree that there is some senescence induction in palbociclib single treatment in figure 3C, but the population of cells is still proliferating and reaches confluence (as shown in colony formation assay in figure 2F and growth assay in figure 3B).

**Reviewer 1, major comment 10:**

The key point of this study is to demonstrate that indisulam treatment can enhance the senescence induction of cells that are resistant to palbociclib. Therefore, the palbociclib pre-treated group is more informative than the parental group as the control.

We would like to thank the reviewer for the comment. This was indeed the reason why we included palbociclib only as a control in all of the experiments.

**Reviewer 1, major comment 11:**

On page 8 lines 170-171, the author mentioned "CDK2 protein levels were not altered compared to individual treatments (Fig. 3E)." In fact, in the left panel of Fig. 3E, the CDK2 protein level in the COMBO group was obviously lower than in the IND group.

The reviewer is correct that the statement could be misinterpreted. We expected that palbociclib treatment would alter CDK2 levels, as CDK4/6 is upstream of CDK2 in the cell cycle. We clarified that CDK2 was not altered when comparing palbociclib alone to the combination treatment in the revised manuscript.

**Reviewer 1, major comment 14:**

It's encouraged to repeat the xenograft experiment in CAL-51 or other cancer cell lines to satisfy the author's claim of multiple cancer types.

It would be valuable to extend the observation to other xenografts indeed. However, we have previously tried to perform these experiments in CAL-51 and SUM159 (see below) and the parental cells were very sensitive to palbociclib treatment in vivo, making it difficult to assess the effect of the combination treatment. The experiment was performed using 100 mg/kg of Palbociclib daily and 5 mg/kg of indisulam 3 times per week.

**Reviewer 1, major comment 15:**

Compared with indisulam, the result in Fig. 5F clearly showed that palbociclib more effectively inhibited CDK2 activity. The explanation was required here.

The reviewer is correct that there is an effect of palbociclib on CDK2 activity. It is important to note that CDK4/6 is upstream of CDK2 in the cell cycle and inhibiting CDK4/6 by palbociclib is therefore expected to reduce the activity of CDK2, as we can observe in figure 5F and 5H. Interestingly, recent publication also shows that CDK4/6 inhibition leads to CDK2 inactivation through p21 (PMID: 34099663).

**Reviewer 1, major comment 16:**

16. On page 11 lines 253-258, the casual relationship here was improper. The author needs to analyze CDK2 activity of overexpressing cyclin H as a rescue experiment in RBM39 knockdown or indisulam treated cells.

We agree that it would be valuable to test if overexpression of cyclin H would rescue the phenotype of the combination treatment. We have tried this experiment multiple times in the past, but could not sustain long term overexpression of cyclin H in the cells. Since the senescence program induced by combination of indisulam and palbociclib requires a long term assay (10 days) and the cells lose their overexpression in 7 days this experiment was not informative. We adjusted the mentioned sentence to make it clear it is a speculation.

**Reviewer 1, major comment 17:**

17. In the right panel of Fig. 5I, RB dephosphorylation by palbociclib was delayed by indisulam in the only SUM139 but not A549. If there was no technical problem, an explanation is required here.

This is indeed an interesting observation. Since these are different cell lines with distinct palbociclib sensitivity it is likely that the dynamics of palbociclib action are different. This was the reason why we included two different time points for the experiment in Figure 5I.

**Reviewer 1, minor comment 1:**

The figure labeling in the main text is messy. For example, Fig. S1B was in the improper sentence, and Fig. 1I-J were missing. Please carefully check this issue.

We apologize for the mistake. We adjusted the labeling in the revised manuscript.

**Reviewer 1, minor comment 3:**

In the Fig S1D upper panel, the cell line should be CAL-51 pLKO but not HCC1806.

We apologize for this mistake. We corrected it in the revised manuscript.

**Reviewer 1, minor comment 4:**

Please do the statistical test for the tumor growth data in Fig. 4A.

We added the statistical test for the tumor volume in the revised manuscript.

**Reviewer 1, minor comment 5:**

The author didn't mention which post hoc test they used after one-way ANOVA.

We added the information about the post hoc test in the figure legend.

**Reviewer 1, minor comment 7:**

The exact concentration labeling was missing in Fig. 5F.

We adjusted the figure in the revised manuscript so that all the concentrations are labeled.

**Reviewer 2, minor comment 1:**

Fig 1 I and J do not exist, but are stated in the text.

This comment is similar to minor comment 1 from Reviewer 1. We apologize for this mistake and adjusted that in the revised manuscript.

**Reviewer 2, minor comment 3:**

The treatment time for indisulam and combo treatment in the in vitro experiments is missing in the figure legends and methods section.

We apologize for the inconsistencies. We included the treatment times in figure legends and methods in the revised manuscript.

**Reviewer 2, minor comment 4:**

How is the proliferation of the CDK2KO clones in A549 and CAL-51 cells in vitro compared to a control?

This comment is similar to major comment 2 from Reviewer 1. We included a statistical test comparing the growth of all the conditions and we note that there was no difference between WT and CDK2 KO cells.

**Reviewer 2, major comment 1:**

How are the CDK2 protein levels upon Palbociclib treatment in all the cell lines?

This comment is similar to major comment 11 from Reviewer 1. We tested CDK2 levels in A549 and SUM159 (Figure 3E). We observed a decrease of total CDK2 upon palbociclib treatment, which is expected as palbociclib inhibits CDK4/6 upstream of CDK2.

**Reviewer 2, major comment 2:**

Fig 3B- The error bars with Palbociclib treatment are extremely high- was the experiment performed only once or are these data really representative for different experiments?

We agree with the reviewer that the error bars in this experiment are high. The experiment was performed 2 independent times with three technical replicates, and one representative experiment is shown in the figure. SUM159 cells are more sensitive to palbociclib monotherapy (see colony formation assay in figure 3A), so there might be some senescent cells present. Those will increase the confluence estimation and the variation between replicates.

**Reviewer 2, major comment 3:**

Were in general the experiments with technical triplicates performed only once or are the data representative for at least 2 different experiments?

All experiments were performed in at least 2 independent biological replicates (except genetic screen and RNA sequencing). We clarified this and included a number of replicates in the figure legends of the revised manuscript.

**Reviewer 2, major comment 5:**

The authors claim that CDK2 levels are not altered but there seems to be a clear reduction with Palbociclib (Figure 3E). A quantification and statistical analysis would help to clarify this issue.

This comment is similar to major comment 11 from Reviewer 1 and major comment 1 from Reviewer 2. With this statement we wanted to point out that there is no difference between palbociclib treatment and the combination in terms of CDK2 abundance. We further clarified this in the text.

**Reviewer 2, major comment 6:**

Figure 3F: Did you include the conditions UT vs Palbo and UT vs IND?

Yes, we performed those comparisons as well. However, we believe it is more informative to compare the effect of the combination to the single treatments.

**Reviewer 2, major comment 8:**

Can you rule out that the indisulam effect on senescence is related to other CDKs e.g CDK7? Please discuss this part in the discussion.

This is an interesting point, as CAK regulates other CDKs as well. We included this point in the discussion of the revised manuscript.

**Reviewer 2, major comment 9:**

Why is Cyclin H only decreased upon 48 hours? Is this in line with the SA-ß-gal induction?

This is an interesting observation. It likely takes some time for the cancer cells in a non-synchronous heterogenous population (due to different phases of the cell cycle) to have the homogeneous effect of the combination treatment. Based on the growth curves in figure 3B, this would be in line with the senescence induction.

**Reviewer 3, minor comment 1:**

The synergy between indisulam and palbociclib is accredited to the inhibition of CDK2 activity via cyclin H downregulation. This claim could be further supported by investigating the effect of indisulam+palbociclib in CDK2-depleted cells. Additionally, cyclin H-depleted cells should be expected to phenocopy the behavior of CDK2-depleted cells.

We think that investigating the effect of the combination on CDK2 KO would not be informative,

because CDK2 KO cells are extremely sensitive to palbociclib alone. Therefore we wouldn’t expect an increase in sensitivity when treating the CDK2 KO cells with the combination. Furthermore, we agree that it would be beneficial to test if loss of cyclin H phenocopies CDK2 loss. Unfortunately, cyclin H is an essential gene and genetic depletion leads to cell death making those experiments technically not possible.

**Reviewer 3, minor comment 2:**

Figure 1G shows the GSEA analysis of sgCDK2+PALBO versus sgCDK2 in CAL-51 cells. The concentration of palbociclib used in this experiment (2,5 mM) is different to the concentration used in the experiment 1F (0,5 mM) which clearly did not induce significant senescence. A concentration of 2,5 mM is likely to induce senescence in CAL-51 cells since it prevents the growth of cell colonies (figure 1D). Therefore, the enriched senescent signatures are likely to be the result of palbociclib cellular effects. To help clarifying this issue SA-Bgal images of CAL-51 cells and CAL-51 sgCDK2 treated with 2,5 mM palbociclib should be presented. Alternatively, the GSEA analysis should be performed either with cells treated with 0,5 mM palbociclib that does not induces senescence in control CAL-51 cells or by comparing sgCDK2+PALBO versus control CAL-51+PALBO.

This comment is similar to the major comment 1 and 3 from Reviewer 1. We agree with the reviewer that this experiment is not so informative, due to the concentration of palbociclib used. We decided to remove this experiment from the manuscript.

**Reviewer 3, minor comment 4:**

The authors refer to the "change in morphology indicative of senescence" (116) and "enlarge size and flat morphological features of cellular senescence" (144) induced by palbociclib in CDK2-depleted cells but do not show any representative image that could allow the evaluation of these changes.

The change in morphology is best visible in SA-B-gal images (Figure 1F, 2D, 2H, 3C, and 5D).

**Reviewer 3, additional minor comment 1:**

Include reference to papers: "Expanding control of the tumor cell cycle with a CDK2/4/6 inhibitor" by Freeman-Cook et al. Cancer Cell, 2021. and "Restoring order at the cell cycle border: Co-targeting CDK4/6 and CDK2". Jeselshohn R et al. Cancer cell, 2021.

We agree it would be valuable to include these references. These publications are recent and were not yet available at the time of manuscript preparation. We included them in the discussion of the revised manuscript.

**Reviewer 3, additional minor comment 2:**

In the legend of figure 2B and 2F the concentration of palbociclib was not specified.

The concentrations used for all colony formation assays are specified in the figure itself.

**Reviewer 3, additional minor comment 3:**

Some figures are not properly referenced:

-Figures 1I and 1J are missing

-Supplementary figures S1B-S1D are not referred to in the text.

-Supplementary figure S1E is referenced as S1B in the text.

We apologize for the mistake and we corrected these mistakes in the revised version of the manuscript.

**Reviewer 3, additional minor comment 4:**

Some figures could be improved:

-Increase size and resolution of figures 1A and S1B.

-Increase size of figures 1H.

We agree with the reviewer. We included an improved quality figure in the revised version.

**Reviewer 3, additional minor comment 5:**

In the discussion section certain sentences are confusing and may lead to misunderstanding or misinterpretation:

"loss of RB leads to resistance to palbociclib....RB deficient cells are still sensitive to knockdown of CDK2. It is therefore likely that the combination of palbociclib and indisulam would still be effective in palbociclib resistant cells." (325-328). These sentences refer to the paper by Pandey et co-workers (reference 52) that propose that inhibition of CDK2 increases senescence by inhibition Phospho-C-MYC with is responsible for acquired resistance to palbociclib and therefore RB negative cells could be sensitive to indisulam. However, they fail to explain why RB-depleted cells are sensitive to palbociclib since intact RB is usually required for the CDK4/6 inhibitor to induce senescence and therefore for palbociclib+indisulam synergy. I would remove these sentences since the authors do not provide experimental evidence of the effect of palbociclib and/or indisulam in RB-deficient cells or the role of c-myc in indisulam+palbociclib synergy.

We agree with the reviewer that this sentence might be misleading. We further explained that the mechanisms of senescence induction in RB deficient cells are poorly understood.

## 4. Description of analyses that authors prefer not to carry out

**Reviewer 1, major comment 7:**

Certain cell lines (ex. A549 and DLD-1) seem to be strongly resistant to palbociclib, which could be further resensitized by CDK2 depletion or inhibition. It's recommended to analyze the correlation between intrinsic CDK2 activity and resistance of palbociclib.

We acknowledge that it would be interesting to see the correlation between CDK2 activity and palbociclib resistance in our cell line panel. However, it has been previously described in breast cancer that increase in Myc, CCNE and CCNA expression leads to increase in CDK2 activity and palbociclib resistance (PMIDs: 32842890, 27020857, 32393270, 33364954, 34520734). We therefore believe this experiment would not contribute to the main conclusions of this paper.

**Reviewer 1, major comment 12:**

According to Fig. 1E, 2G, S4A, and S4B, CDK2 activity or expression was required for in vivo tumor growth but not in vitro cell growth. However, the treatment of indisulam only slightly delayed the tumor growth. The author should perform the CDK2 activity assay on the in vivo tumor cells to prove that the administration of indisulam indeed inhibited CDK2 function.

We acknowledge that it would be interesting to understand CDK2 activity *in vivo* after indisulam treatment. Unfortunately the CDK2 reporter we used *in vitro* is not suitable for

CDK2 activity experiments *in vivo*.

**Reviewer 1, major comment 13:**

There is no evidence supporting that COMBO treatment induced tumor senescence in vivo and resensitized tumor to senolytic therapy. Ki67low and p21high related to cell cycle arrest but not permanent arrest, which is the definition of senescence. Thus, the results in Fig.4 only illustrated that the combination of CDK2 inhibitor and CDK4/6 inhibitor repressed proliferation and tumor growth, which were not impactful enough. The author should suspend the treatments and then confirm whether the tumor cells stay in the resting phase. On the other hand, the senolytic treatment (ABT-263) should be also performed in the xenograft model.

We agree with the reviewer that Ki67 and p21 are not exclusive for senescence. However, we believe that a drug washout experiment would not be informative as it is unexpected for cancer cell senescence to be homogeneous in vivo and therefore will not lead to a homogenous arrest in tumor growth. We would also like to point out that there is ample evidence in the literature that senescence is not strictly irreversible (for example: PMIDs 12912919; 30803994; 12551891), so a re-growth after wash-out would not provide strict evidence that the cells were not senescent under drug treatment. We agree with the reviewer that it would be interesting to expand the observation on senolytic drugs to in vivo. However, we feel this is outside the scope of this paper.

**Reviewer 1, minor comment 2:**

The author should quantify protein amount for confirming knockdown and overexpression but not mRNA.

As we already show protein amounts for CRISPR based experiments (Figure 1C-F, Figure 2A-H) we believe it is not necessary for the shRNA based experiments. We could include these data if the editors feel strongly that this should be included.

**Reviewer 2, minor comment 2:**

Is there a senescence signature in SUM159 upon loss of CDK2? If not, please discuss the (possible) discrepancy to the other cell lines.

CDK2 KO SUM159 cells are still proliferating (as in Figure 2B,C) so we do not expect them to be senescent. We tested CDK2 KO cells treated with palbociclib and observed proliferation arrest (Figure 2B and C) and increase in SA-B-gal staining (Figure 2D). We believe these markers are sufficient to show senescence and we did not perform RNA sequencing of those samples.
