# Supplementary material for: Indisulam synergizes with palbociclib to induce senescence through inhibition of CDK2 kinase activity
Source: PLoS One. 2022 Sep 6;17(9):e0273182. doi: 10.1371/journal.pone.0273182 (PMC9447877; doi:10.1371/journal.pone.0273182)
Supplement: Attachment — Submitted filename: Rebuttal PLOS one_1.2.docx [file pone.0273182.s005.docx]

PONE-D-21-35335

Pogacar et al

We would like to thank the reviewers for their detailed reading of the manuscript and the insightful comments. We believe that the revised manuscript adequately addresses the reviewers’ questions.

1. Is the manuscript technically sound, and do the data support the conclusions?

The manuscript must describe a technically sound piece of scientific research with data that supports the conclusions. Experiments must have been conducted rigorously, with appropriate controls, replication, and sample sizes. The conclusions must be drawn appropriately based on the data presented.

Reviewer #1: Yes

Reviewer #2: Partly

2. Has the statistical analysis been performed appropriately and rigorously?

Reviewer #1: Yes

Reviewer #2: No

In order to improve this, we included quantification and statistical testing on SA-β-gal stainings (Figure 1G, 2E and J, 3D, 5E, S1F, S3D and G). Furthermore, we performed statistical testing on all proliferation experiments (Figure 1E, 2C and H, 3B, 5C, S1D, S3B) and growth curves of the *in vivo* experiment (Figure 4A).

3. Have the authors made all data underlying the findings in their manuscript fully available?

The [PLOS Data policy](https://eur04.safelinks.protection.outlook.com/?url=http%3A%2F%2Fwww.plosone.org%2Fstatic%2Fpolicies.action%23sharing&data=04%7C01%7Cr.ld.oliveira%40amsterdamumc.nl%7C2081b1d594f54f57b3cd08d9c662e62b%7C68dfab1a11bb4cc6beb528d756984fb6%7C0%7C0%7C637758951209380816%7CUnknown%7CTWFpbGZsb3d8eyJWIjoiMC4wLjAwMDAiLCJQIjoiV2luMzIiLCJBTiI6Ik1haWwiLCJXVCI6Mn0%3D%7C3000&sdata=FSp5%2B1yvZahZNH5zjGM8OKWDT8QnGRtyfRY9aTuHKHk%3D&reserved=0) requires authors to make all data underlying the findings described in their manuscript fully available without restriction, with rare exception (please refer to the Data Availability Statement in the manuscript PDF file). The data should be provided as part of the manuscript or its supporting information, or deposited to a public repository. For example, in addition to summary statistics, the data points behind means, medians and variance measures should be available. If there are restrictions on publicly sharing data—e.g. participant privacy or use of data from a third party—those must be specified.

Reviewer #1: Yes

Reviewer #2: Yes

4. Is the manuscript presented in an intelligible fashion and written in standard English?

PLOS ONE does not copyedit accepted manuscripts, so the language in submitted articles must be clear, correct, and unambiguous. Any typographical or grammatical errors should be corrected at revision, so please note any specific errors here.

Reviewer #1: Yes

Reviewer #2: Yes

5. Review Comments to the Author

Reviewer #1: Authors tried to evaluate the induction of senescence in cancer cells for a new therapeutic strategy. They found that blocking CDK2 by indisulam, a novel sulfonamide anticancer agent (N-(3-chloro-7-indolyl)-1,4-benzenedisulfonamide, E7070), would enhance senescence induction by palbociclib, a CDK4/6 inhibitor approved for treatment of metastatic breast cancer, in triple negative breast cancer (TNBC) cells, various cell lines and lung cancer xenografts. Thus, they concluded that combined treatment with palbociclib and indisulam induced a senescence program and sensitized cells to senolytic therapy, illustrating that inhibition of CDK2 through indisulam treatment can enhance senescence induction by CDK4/6 inhibition. Whole manuscript clearly shows the good rationale, methods, results and figure illustrations. However, there is no space between word and citation parenthesis in the text, which should be correctly reformatted.

We were happy to hear the reviewer agrees that the rationale and methods of our findings are relevant to the field. Furthermore, we have adapted the formatting of citations as suggested.

Reviewer #2: Major concerns:

1. Previous report demonstrated that indisulam (E7070) inhibited the phosphorylation of pRb, decreased expressions of cyclin A, B1, CDK2, and CDC2 proteins, and suppressed CDK2 catalytic activity with the induction of p53 and p21 proteins in A549 cells but not in A549/ER cells (Invest New Drugs 2001;19:219-27). Therefore, the combined therapeutic benefit of indisulam and palbociclib still did not prove due to the inhibitory effect of CDK2 by indisulam, the off-target effect of indisulam may also play a role to contribute to this combined benefit.

We agree with the reviewer that indisulam likely has other effects in addition to CDK2 inhibition. We propose that indisulam is an indirect CDK2 inhibitor, as overexpression of CDK2 leads to partial rescue of senescence induction by palbociclib and indisulam (figure 5N).Moreover, we included a comment about CDK2 independent effects of indisulam in the discussion (line 268-269).

2. Looking at the research, there is no direct evidence to prove that indisulam synergizes with palbociclib to induce senescence through inhibition of CDK2 kinase activity (Title). First, the author did not present evidence that indisulam inhibits CDK2 kinase activity. Secondly, the author has not proved that indisulam induces senescence by inhibiting CDK2 kinase activity.

We studied the effects of indisulam treatment on CDK2 activity using a CDK2 reporter in figure 5G and I. We observed that treatment with indisulam prevented CDK2 activation in cells where CDK2 was initially inactive. This suggests that indisulam indirectly prevents CDK2 activity. Secondly, overexpression of CDK2 rescues the senescence induction of cells treated with palbociclib and indisulam as shown in figure 5N. We believe that these experiments support our conclusion that senescence induction by palbociclib and indisulam is mediated by CDK2 activity.

3. There is not enough evidence to prove that indisulam is a CDK2 inhibitor in the present study. In fact, the results show that palbociclib alone has a more pronounced inhibitory effect on CDK2 (Figure 3E). The relevant issues need to be clarified.

- We believe that indisulam is an indirect CDK2 activity, preventing CDK2 activation and not changing the abundance of the protein. We would therefore not expect to observe a decrease in CDK2 total protein when treating cells with indisulam (Figure 3F). On the other hand, we do observe reduction in active CDK2 in indisulam treated cells (Figure 5N) which indicates that indisulam prevents CDK2 activation. Furthermore, palbociclib inhibits CDK4/6 which is upstream of CDK2 in the cell cycle and has been previously demonstrated to influence CDK2 abundance (PMID: 27020857). It is therefore not unexpected that total levels of CDK2 are reduced in palbociclib treated cells, which we also observed in RNA seq experiment. We added a comment clarifying this in the text (line 171-173).

4. Authors indicated that treating cells with indisulam led to downregulation of cyclin H, which prevented CDK2 activation. However, indisulam did not reduce cyclin H levels till 48 hours of treatment (Figure 5I), but CDK2 activity tests are only analyzed within 24 hours. Such experimental design and results cannot explain that treating cells with indisulam led to downregulation of cyclin H, which prevented CDK2 activation.

We agree that there are differences between the western blot and imaging experiments. The microscopy experiments were performed in an acute setting: a shorter time interval and with much higher concentration of indisulam. It is technically not possible to perform these experiments with longer time points due to effects on cell viability. On the other hand, the Western blot experiment was performed with lower concentration of indisulam which could explain the difference in timing of cyclin H downregulation. Additionally, a small decrease of cyclin H might not be obvious on Western blot, but could already result in lower CDK2 activation.

5. Authors mentioned that combined treatment with palbociclib and indisulam induced a senescence program and sensitized cells to senolytic therapy. According to the result, this event is only found in A549 cells, but not SUM159 cells (Figure 3D). The relevant issues need to be clarified.

We agree that the effect of ABT-263 in SUM159 was not convincing. We believe the senescence induction was less efficient in that experiment. We therefore repeated the experiment while monitoring senescence induction and now observed sensitisation of senescent cells to ABT263 treatment. We have changed the figure to include new data (Figure 3E).

6. There is no evidence to support that COMBO therapy induces tumor senescence in vivo and resensitizes tumors to senolytic therapy, and relevant markers need to be checked.

Senescence characterisation *in vivo* is challenging due to heterogeneity of senescence induction *in vivo*. As the cell line we used for the xenograft model (A549) is p16 deficient we chose to characterise p21 and Ki67 instead. We agree that sensitivity to senolytic therapy *in vivo* would be interesting, but we believe this experiment is out of the scope of this project.
