# Supplementary material for: Indisulam synergizes with palbociclib to induce senescence through inhibition of CDK2 kinase activity
Source: PLoS One. 2022 Sep 6;17(9):e0273182. doi: 10.1371/journal.pone.0273182 (PMC9447877; doi:10.1371/journal.pone.0273182)
Supplement: Attachment — Submitted filename: Pogacar_Rebuttal.docx [file pone.0273182.s006.docx]

We would like to thank the reviewers for their careful reading of our manuscript and constructive comments. We believe that this second round of revisions further improved the quality of the manuscript and we hope you agree that we adequately addressed the reviewer’s comments.

REBUTTAL PLOS ONE

Reviewer #3: In this manuscript by Ziva Pogacar et al., the authors tried to broaden clinical utility of CDK4/6 inhibitor in some cancers that CDK4/6 inhibitors are limited used. They performed functional shRNA screens in palbociclib resistant cells and found knockdown of CDK2 makes the cells more sensitive to palbociclib. The experiments are performed in multiple cell lines and also confirmed in vivo, overall, the experimental designs are rigor, the conclusions are supported by solid data. There are a few comments needed to be addressed:

We were happy to hear the reviewer agrees that our experiments support the conclusions and our findings are relevant to the field.

1. Indisulam was used as a CDK2 inhibitor in this paper, so I prefer the author show some background of indisulam that used as CDK2 inhibitor in the introduction part.

We agree with the reviewer that it is important to introduce previous literature on indisulam as a CDK2 inhibitor. We added this information and a citation to the introduction (line 88-89).

2. Fig. 1C, is the CAL-51-CKD2 knockout cell line monoclonal cell line or polyclonal cell line, why there are still bands in the two CAL-51-CKD2 knockout cell lines.

Those cell lines are indeed polyclonal, which is why there is some background CDK2 expression visible on the Western blot. We have clarified that the lines are polyclonal in the figure legend (line 382).

3. For one of reviewers’ questions: There is no direct evidence to prove that indisulam synergizes with palbociclib to induce senescence through inhibition of CDK2 kinase activity (Title). The author did not present evidence that indisulam inhibits CDK2 kinase activity. According to the author’s response, the author didn’t provide direct evidence to confirm CDK2 kinase activity was prevented by indisulam and Palbociclib. To address this question, the author should at least test the expression of phosphorylation of CDK2 after treated with indisulam or combine with palbociclib.

We agree with the reviewer that the data on CDK2 activity is indirect. However, our results on CDK2 reporter indicate that indisulam prevents activation of CDK2. The experiment assessing endogenous CDK2 phosphorylation on Thr160 (activation) is unfortunately technically not feasible, because the antibody is not specific to CDK2 but also recognises CDK1 phosphorylation on Thr161.

Reviewer #4: In this manuscript, the author found that indisulam prevented CDK2 activation and can synergistically induce senescence when it was combined with Palbociclib, a CDK4/6 inhibitor. I have several comments for the author to consider.

1. The author declared a synergy between indisulam and palbociclib in the induction of senescence, but no related data to show this. The author may consider using combination index (CI) values or other similar indices to exhibit synergistic effects for related detections (Control, PALBO, IND, COMBO).

We agree with the reviewer that combination indexes might be useful when showing synergistic effects on cell viability of two drugs. However, in our case the phenotype we observe upon combination is senescence not cell death. Senescent cells are known to change their metabolism so a metabolic read-out (such as resazurin assay) typically used to calculate combination indices is not reliable in this case. We therefore opted for proliferation assays to show the effect of combination treatment to cell growth and show full growth arrest upon combination treatment, while cells grow out in individual treatments (Figure 3B and S3B).

2. It would be better if the authors had data for senolytic drug ABT-263 assays with colorectal cells.

We agree with the reviewer that it would be beneficial to show response to ABT-263 in additional cell lines. We have added data on DLD-1 and RKO parental and senescent cells treated with ABT-263 to Supplemental figure 3E.

3. For detections with PALBO, IND, and COMBO, the author may consider applying 2-factor ANOVA or 3-factor ANOVA (if having a time effect) analysis.

Thank you for your comment. To analyze the proliferation experiments we used 2-factor ANOVA for genetic experiments (Figure 1E, 2C, 2H and S1D) where one factor is genotype and another is treatment. For drug treatment experiments we used 1-factor ANOVA, as we only have one factor: treatment and we analyzed every cell line separately. We chose to only analyze the end points of the proliferation assay to allow easier interpretation together with colony formation assays and SA-Bgal stainings which are both end-point assays.

4. For Fig.3D and similar figure panels in other figures, the authors only compared all other groups to the combination groups. Please clarify or modify.

We agree with the reviewer that the comparison to combination group should be further explained. As palbociclib is already known to be a weak senescence inducer, we believe that comparing each treatment to untreated control would not be so informative. We aimed to compare the individual treatments to the combination group to show that combination is superior in senescence induction, which is why we compared each treatment group to the combination. We have clarified the choice of analysis in the methods section (line 857-859).

5. It would be better if the author could quantify protein expression for Fig.3F and 5J by looking at the loading controls. The changing trends will be easy to distinguish accordingly.

We agree that the changes in protein abundance are sometimes not very obvious. We have therefore quantified the protein abundance as suggested by the reviewer and added them to the revised manuscript and figures (see Figure 3G and Supplemental Figure 5B).

6. Please add statistical significance for Figures S4A and B

We agree with the reviewer that statistical analysis would help the interpretation of the data in Figure S4A and B. We performed the analysis and added them to the revised manuscript (Figure S4A and B).

7. The authors only provide statistical significance for the endpoint in Figure 4A. Please modify.

The reviewer is correct that we analysed tumor volumes at the end point of the experiment. We decided for that as samples for stainings were taken at the same time point, so we believe this allows for more relevant conclusions and comparisons between different groups.

8. For Figures 5C, 5D, 5F, 5H, 5J, 5K, and 5N, the authors frequently changed the concentrations of PALBO and/or IND used in different detections compared to previous assays. Please clarify each of them.

The reviewer is correct that we used different concentrations in different experiments. We typically used lower concentrations for long term experiments, such as proliferation assays, colony formation assays and B-gal stainings (Figure 5C, D, N) and higher concentrations for short term experiments such as imaging experiments with CKD2 reporter, short-term Western blots and FACS (Figure 5G, H, I, J). We clarified the use of concentrations between different experiments in the results section of the revised manuscript (line 234-235).

9. The data is not strong enough to support that indisulam-induced CCNH downregulation prevents CDK2 activation through the CAK-complex inactivation. Please consider providing more explanations in the discussion section.

We agree with the reviewer that other factors might play a role in senescence induction. We added an additional explanation to the discussion (lines 326-328).

10. In the legend of Figure S1, does “n = 2” means two biological replicates, or was the experiment repeated twice? It is confusing. Please check all legends for similar cases.

We agree with the reviewer that the figure legends could be confusing. We have adapted all figure legends to clarify independent experiments and technical replicates.

11. Please check if one-way ANOVA or two-way ANOVA analysis were used correctly. For example, Figure 1G, Figure 3D, etc.

This comment is similar to comment no.3. As explained above, we performed two-way ANOVA when analysing genetic experiments (one factor is genotype, another is treatment) - for example Figure 1G, S1F, 2E, 2J and one way ANOVA for drug experiments (one factor is treatment) for example Figure 3D, S3D.

12. The authors may consider providing an extra figure to better summarize mechanisms or regulatory pathways found.

We agree with the reviewer that a model can clarify the pathways involved. We have added a model to Figure 5O.

13. line 166-173, please cite figure panels appropriately.

We have re-checked and adjusted citing of the figures where needed.
